# Measuring the Elasticity of Poly‐l‐Proline Helices with Terahertz Spectroscopy

**DOI:** 10.1002/anie.201602268

**Published:** 2016-04-28

**Authors:** Michael T. Ruggiero, Juraj Sibik, Roberto Orlando, J. Axel Zeitler, Timothy M. Korter

**Affiliations:** ^1^ Department of ChemistrySyracuse University1-014 Center for Science and TechnologySyracuseNY13244-4100USA; ^2^ Department of Chemical Engineering and BiotechnologyUniversity of CambridgeNew Museums SitePembroke StreetCambridgeCB2 3RAUK; ^3^ Dipartimento di Chimica and Centre of Excellence Nanostructured Interfaces and SurfacesUniversità di Torinovia Giuria 510125TorinoItaly; ^4^ F. Hoffmann-La Roche AGKonzern-HauptsitzGrenzacherstrasse 1244070BaselSwitzerland

**Keywords:** biopolymers, elasticity, polyproline, proteins, terahertz spectroscopy

## Abstract

The rigidity of poly‐l‐proline is an important contributor to the stability of many protein secondary structures, where it has been shown to strongly influence bulk flexibility. The experimental Young's moduli of two known poly‐l‐proline helical forms, right‐handed all‐*cis* (Form I) and left‐handed all‐*trans* (Form II), were determined in the crystalline state by using an approach that combines terahertz time‐domain spectroscopy, X‐ray diffraction, and solid‐state density functional theory. Contrary to expectations, the helices were found to be considerably less rigid than many other natural and synthetic polymers, as well as differing greatly from each other, with Young's moduli of 4.9 and 9.6 GPa for Forms I and II, respectively.

The ability of a protein to maintain proper secondary structure^1^ is reflected in its elasticity,^2^ which represents the tendency of the system to structurally deform under external forces. Several studies have invoked elasticity to explain the origins of observed protein properties, including structural stability,^3^ mechanical strength,^4^ and catalytic activity.^5^ Despite the great relevance of elasticity, its quantification in large biomolecules has proven to be an elusive goal owing to the difficulties associated with measuring stress–strain curves for these materials.^6^ Common methods for studying protein flexibility include X‐ray crystallography and NMR spectroscopy,^7^ but neither is able to provide specific values for the elastic parameters, and only general inferences can be drawn. To overcome the experimental limitations, several indirect approaches have been developed that attempt to relate amino acid sequences in various domains to bulk elasticity, but these often rely on simple empirical data (i.e., packing density and intermolecular contacts), rather than actual stress–strain probe measurements.5a, 8 A promising alternative experimental technique is vibrational spectroscopy because it offers the advantage of being able to probe the stress (energy) and resulting strain (motion) of particular vibrational modes.^9^ A drawback is that traditional vibrational methods (e.g., mid‐infrared spectroscopy^10^) probe only the motions localized to individual bonds. This may yield elastic information about the specific bond, but does not provide knowledge of the sample in its entirety. Therefore, different types of vibrations must be considered.

Terahertz time‐domain spectroscopy (THz‐TDS) is a powerful tool for accessing sub‐150 cm^−1^ vibrational modes that involve large‐amplitude global molecular motions, and it has proven useful for characterizing biomolecules such as cellulose and DNA.^11^ These low‐frequency motions include both external (rotation and translation) and internal (torsion) vibrations of condensed‐phase sample components, meaning that both the bulk and localized stress–strain relationships can be simultaneously explored. The vibrational force constants determined through THz‐TDS are a direct measure of the elastic properties of the studied material, yielding immediately useful elastic constants such as the Young's modulus through classical relationships to Hooke's law. This approach was used in this work to characterize the two helical conformations of the poly‐l‐proline polypeptide and evaluate its rigidity as compared to other polymers.

Poly‐l‐proline, a component of collagen,^12^ is considered to be a rigid peptide sequence and it is often found in proteins where it is believed to add mechanical stability to secondary structure.^13^ Proline is unique amongst naturally occurring amino acids as the only residue able to readily form both *cis* and *trans* configurations about its peptide bond linkages,^14^ thereby permitting two different helical structures to exist for poly‐l‐proline.^15^ The all‐*cis* right‐handed helix (Form I, PP‐I) is tightly wound,^16^ while the all‐*trans* left‐handed helix (Form II, PP‐II) adopts a less dense geometry (Figure 1).^17^ The availability of these similar, yet fundamentally different, poly‐l‐proline helices makes them excellent choices for exploring the connection between molecular structure, low‐frequency vibrational motions, and bulk elastic constants.

**Figure 1:**
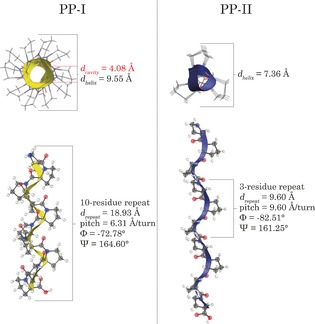
Structures of ten‐residue fragments of the poly‐l‐proline isomers PP‐I and PP‐II. The distance per crystallographic repeat (*d*_repeat_), pitch (distance per helical turn), the two Ramachandran angles (*φ* and Ψ), as well as the diameter of the helix and cavity (for PP‐I only, not applicable to PP‐II) determined through ss‐DFT calculations are shown.^32^

While the rigidity of poly‐l‐proline chains has been explored within protein structures,13c no studies of the actual elasticity have been performed. This dearth of information is in part due to a lack of atomic‐level structural data for either helix. Herein, the terahertz vibrations of PP‐I and PP‐II were assigned and analyzed by using structures determined from a combination of experimental powder X‐ray diffraction (PXRD) and solid‐state density functional theory (ss‐DFT) calculations. Collectively, these techniques enable quantification of the elastic properties of this large biopolymer.

The low‐temperature (78 K) THz‐TDS vibrational spectra (Figure 2) of solid PP‐I and PP‐II (1–10 kDa; for experimental details, see the Supporting Information) were acquired over a 20–150 cm^−1^ (0.6–4.5 THz, 7.6 GHz resolution) spectral window with a Cherenkov‐radiation based source,^18^ thereby permitting features to be identified beyond the reach of more commonly available instruments.^19^ The THz‐TDS spectra of both samples contain distinct features that are specific to the conformation of each poly‐l‐proline helix and also to the three‐dimensional arrangement of the helices in the solid state. The analysis of the vibrational data began with full redetermination of the complete crystal structures of both PP‐I and PP‐II.

**Figure 2:**
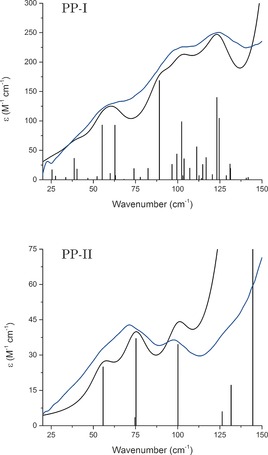
Low‐temperature (78 K) THz‐TDS spectra of PP‐I and PP‐II (blue), overlaid with simulated vibrational spectra (black).

Powder X‐ray diffraction measurements (90 K) were performed on both samples (Figure 3), and the results revealed numerous Bragg reflections unique to each solid, surpassing the quality of those previously reported.^16^, ^17^ The samples were free from any noticeable cross‐contamination, as evidenced by the lack of reflections from the complementary form in both patterns. Despite the high‐quality PXRD patterns, such data alone were not sufficient for complete structural determinations with atomic precision, and utilization of computational methods was necessary to arrive at detailed solutions.

**Figure 3:**
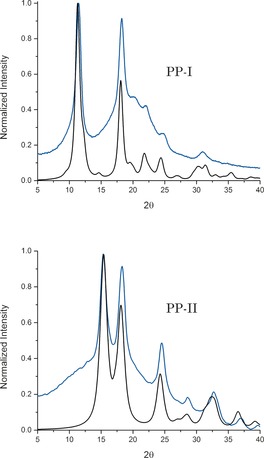
Experimental (blue) and calculated (black) PXRD patterns of the two forms of solid‐state poly‐l‐proline.

In the case of PP‐I, initial crystal structures were constructed using the previously published interatomic distances and angles,^16^ but with the solid‐state packing arrangements and strand orientations varied (for details, see the Supporting Information). After full ss‐DFT optimization, the PP‐I crystal was found to have monoclinic *P*2_1_ symmetry in agreement with estimates made by Shmueli and Traub (Figure 4).^20^ The unit cell contains a single all‐*cis* poly‐l‐proline helix that makes three complete turns over the course of 10 residues, with the helical axis corresponding to the crystallographic *b*‐axis. This arrangement results in an infinite matrix of neighboring helices oriented parallel to each other and extending throughout the entire crystalline solid.

**Figure 4:**
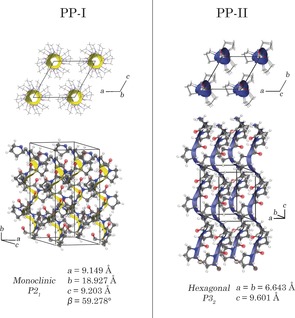
Solid‐state packing structures (two views) and crystallographic parameters of PP‐I and PP‐II.^32^

The structure of PP‐II is similar to that reported previously,^17^ with the most obvious advancement being inclusion of hydrogen atom positions. PP‐II crystallizes in the hexagonal *P*3_2_ space group, and similar to the PP‐I structure, the unit cell contains a single all‐*trans* helix with three proline residues corresponding to a single helical turn (Figure 4). The PP‐II helices are arranged parallel to each other in order to minimize void space, but the more extended PP‐II helix enables more efficient packing than PP‐I, thereby maximizing London dispersion interactions. This is particularly important because both poly‐l‐proline structures lack any hydrogen bond donors, meaning that interhelix interactions are due entirely to London dispersion and dipolar forces.

With the two poly‐l‐proline structures solved, calculation of the vibrational eigenvectors and eigenvalues could be performed to enable assignment of specific modes for determination of the elastic properties of the helices. Considering PP‐II first, where the higher crystalline symmetry results in a lower number of IR‐active vibrational modes, a correlation between experiment and theory can be observed (Figure 2, bottom). The lower symmetry of PP‐I results in a far greater number of IR‐active vibrational modes and a higher spectral density in the low‐frequency region, but the major contributing modes can still be assigned. The sub‐150 cm^−1^ vibrational motions of both forms, determined by visualization of the eigenvector displacements, are primarily rotations and torsions of the pyrrolidine rings that result in complex spring‐like elongation and contraction of the helix (Figure 5). Specifically, the 68.15 cm^−1^ mode (exp. 66.6 cm^−1^) in PP‐I and the 100.10 cm^−1^ mode in PP‐II (exp. 98.1 cm^−1^) are most representative of the prototypical helical compression–extension motion, thus making them prime candidates for Young's modulus determination through the use of vibrational force constants.

**Figure 5:**
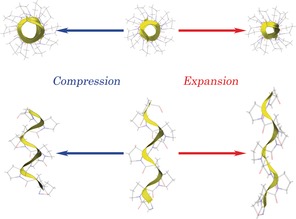
Visualization of the eigenvector displacements of the 68.15 cm^−1^ (exp. 66.6 cm^−1^) vibration in PP‐I, showing the observed spring‐type motion.

Young's modulus (*Y*) is used to describe the rigidity of solids, with a higher value being indicative of a more rigid structure (*Y*_rubber_≈0.01 GPa,^21^ *Y*_iron bar_≈200 GPa^22^). The equation for Young's modulus relates the stress $\left(\sigma \right)$ to the strain $\left(ɛ\right)$ and is commonly calculated from the slope of an experimental stress–strain curve,

(1) $$Y=\frac{\sigma }{ɛ}=\frac{FL_{0}}{A_{0}\Delta L}$$

where *F* is the force exerted on the material, $L_{0}$ and $A_{0}$ are the equilibrium length and area of the material, respectively, and ΔL is the change in the equilibrium length of the sample. It becomes clear that rearranging the equation for Young's modulus results in a form of Hooke's law,
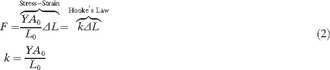



which is a valid assumption when considering small stresses and strains.^23^ The connection between Hooke's law and Young's modulus can be leveraged through vibrational spectroscopy, since it relates the vibrational frequency (ν ) of a harmonic oscillator with a reduced mass (μ ), to an analogue of the classical force constant (k ),

(2) $$\nu =\frac{1}{2\pi }\sqrt{\frac{k}{\mu }}=\frac{1}{2\pi }\sqrt{\frac{{YA}_{0}}{L_{0}}\frac{1}{\mu }}$$

The low‐frequency motions accessible by THz‐TDS are large‐amplitude vibrations of the entire bulk structure, and therefore can be used to determine Young's modulus for the solid.

Using the observed terahertz frequencies and calculated μ values, the experimental force constants could be determined for the two assigned spring modes, thereby ultimately yielding the Young's moduli of the different polyproline helices (Table 1). The elastic properties of the two polyproline helices were verified computationally by using ab initio methods (not through vibrational force constants).^24^ The results (Table 1) show that the values for Young's moduli calculated entirely from first principles are in very good agreement with those determined by using the experimental terahertz vibrational frequencies. Additionally, as an independent check of the applied theory, the Young's modulus of crystalline polyethylene was calculated by using the same methods, and the results (*Y*=14.63 GPa) matched well with previously published data^25^ (*Y*=15.8 GPa).

**Table 1:** Vibrational frequencies (cm^−1^), force constants (N m^−^), reduced mass (Da), and Young's moduli (GPa) determined for PP‐I and PP‐II crystals from the terahertz data and first principles calculations.

	Experimental			Calculated				Ab initio
	$\tilde{\nu }$	*k* ^[a]^	*Y*	$\tilde{\nu }$	*k*	*µ*	*Y*	*Y*
PP‐I	66.6	1.9	4.9±0.2	68.15	1.94	7.08	5.04	5.06
PP‐II	98.1	3.8	9.6±0.1	100.11	3.91	6.62	9.82	10.57

[a] Derived using calculated μ values.

Contrary to suggestions in the literature,^26^ the elasticity results indicate that poly‐l‐proline is actually considerably less rigid than many other common polymeric materials,^25^, ^27^ although it is more rigid than poly‐l‐alanine (Table 2).^28^ The significantly different rigidities of the two forms of poly‐l‐proline, with PP‐II showing an approximately 96 % larger Young's modulus than PP‐I, is due to differences in the peptide bond geometries between the two structures. The near orthogonal orientation of the *cis* peptide bond with respect to the helical axis in PP‐I means that any change in it leads to a large alteration in the overall helical length. This was confirmed computationally by comparing the geometries of the two helices after manually increasing the helical axes from their equilibrium lengths by 10 % and subsequently allowing the structures to relax within the constraint of fixed helical length. The results showed that both the covalent bond lengths and dihedral angles were distorted in PP‐I (average absolute change per Å of distortion of 0.002 Å and 2.678°, respectively) by a much smaller degree than PP‐II (average absolute change per Å of distortion of 0.024 Å and 13.08°, respectively), despite the same relative change in helical length. Additionally, the calculations provided some insight into the energy required for conversion of the more stable PP‐II structure into the PP‐I form (Δ*G*_298K_=4.39 kJ mol^−1^ per residue). The 10 % elongation of the PP‐II helix and concomitant dihedral angle changes resulted in an energy increase within the polypeptide of 10.02 kJ mol^−1^ per Å of distortion, which serves as a preliminary indicator of the barrier opposing the formation of PP‐I. These results are consistent with previous studies of the poly‐l‐proline transformation, which found that large activation energy barriers exist along the conversion coordinate.^15^, ^29^

**Table 2:** List of Young's moduli (GPa) for crystalline polymeric systems.

Polymer	Young's Modulus
Poly‐l‐alanine^28^	2.4–2.9
Poly‐l‐proline Form I^[a]^	4.9±0.2
Poly‐l‐proline Form II^[a]^	9.6±0.1
Poly(methyl methacrylate) Single Helix27a	11
Polyethylene^25^	15.8
Poly(methyl methacrylate) Double Helix27a	19
Collagen27c	21
Cellulose27d	25
Poly‐l‐glycine27b, 31	42

[a] This work.

The measurement of biopolymer elasticity through a combined approach of THz‐TDS experiments and ss‐DFT simulations enables quantification of molecular rigidities to be achieved in a relatively straightforward way. This methodology has yielded the previously unmeasured Young's moduli of the widespread poly‐l‐proline polypeptide in both its helical forms, and revealed them to be considerably more elastic than expected. This prompts contemplation of their role as an analytical standard for rigidity.^30^ This method could, in principle, be applied to other biological systems of any phase; however an ordered crystalline environment greatly facilitates interpretation of the spectral data. Ultimately, reliable quantification of biomolecular elasticity promotes a complete understanding of the factors affecting protein stability and the mechanisms associated with structural change.^33^

*In memory of Roberto Orlando*

## Supporting information

As a service to our authors and readers, this journal provides supporting information supplied by the authors. Such materials are peer reviewed and may be re‐organized for online delivery, but are not copy‐edited or typeset. Technical support issues arising from supporting information (other than missing files) should be addressed to the authors.

Supplementary
