# Supplementary material for: Measuring the Elasticity of Poly‐l‐Proline Helices with Terahertz Spectroscopy
Source: Angew Chem Int Ed Engl. 2016 Apr 28;55(24):6877–81. doi: 10.1002/anie.201602268 (PMC4999051; doi:10.1002/anie.201602268)
Supplement: Supplementary file 1 — Supplementary [file ANIE-55-6877-s001.pdf]

Angewandte Chemie
Eine Zeitschrift der Gesellschaft Deutscher Chemiker

Supporting Information

Measuring the Elasticity of Poly-L-Proline Helices with Terahertz Spectroscopy

Michael T. Ruggiero, Juraj Sibik, Roberto Orlando[†]*, J. Axel Zeitler, and Timothy M. Korter**

anie_201602268_sm_miscellaneous_information.pdf

Supporting Information

A. Experimental Methods **2**

B. Structure Determination Details **4**

C. Gibbs Free Energy Curves **6**

D. Spectral Deconvolution Analysis Results **8**

E. PP-I CIF **15**

F. PP-II CIF **17**

G. References **18**

A. Experimental Methods

Powder X-ray diffraction (PXRD) measurements were performed using a Bruker KAPPA APEX DUO diffractometer equipped with a CCD detector. Data was acquired at 90 K using monochromated Cu Kα radiation (λ = 1.54056 Å). The diffraction patterns were created using the Bruker APEX2 software package.[1] Calculated PXRD patterns were generated using Mercury.[2]

Poly-L-proline was purchased from Sigma Aldrich (1 kDa – 10 kDa), and the initial PXRD patterns showed evidence of a largely amorphous material. PP-I was synthesized by dissolving the polyproline in a 1:9 formic acid to *n*-propanol solution and allowing the mixture to stand for three days, after which an equal volume of diethyl ether was added and subsequently allowed to evaporate. Upon evaporation, ethanol was added and the solution was stirred under gentle heating, resulting in obtaining microcrystalline PP-I. PP-II was synthesized by dissolving polyproline in a 1:3 formic acid to water mixture, and allowing the solution to evaporate while stirring. The PXRD patterns did not show any detectable sign of contamination from the other helical form in both cases.

Samples were prepared for terahertz spectroscopy by mixing poly-L-proline with polytetrafluoroethylene (PTFE, ~3.5 % w/w) and pressing into13mm diameter pellets. The terahertz absorption spectra were obtained using a commercial Advantest TAS7500TS spectrometer in the 20-150 cm^{-1} range. The broadband terahertz radiation was generated using an Advantest TAS1130 source module that contained a lithium niobate ($LiNbO_3$) nonlinear crystal, and was detected using an Advantest TAS1230 detector module. The spectra were treated using the empirical mode deconvolution (EMD) method[3,4] to remove oscillations that result from

reflections of the terahertz pulse that occurred while scanning over large delay distances, and as an example, the uncorrected PP-I spectrum is shown in **Figure S.1**.

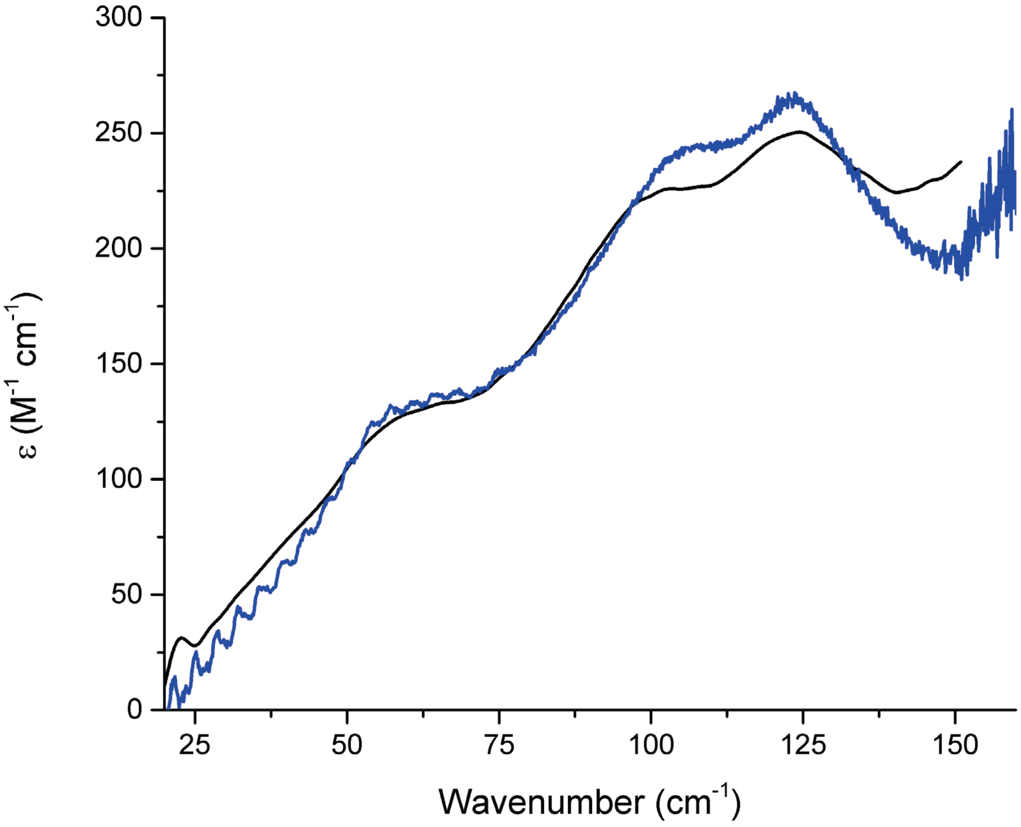

Figure S.1. Unmodified (blue) and corrected (black) terahertz spectra of PP-I, highlighting the etalon interference oscillations that were ultimately removed using the empirical mode deconvolution method.

The quantum mechanical simulations were performed using a developmental version of the CRYSTAL14 software package.[5] The all-electron split-valence double-ζ def2-SVP[6] basis set and Becke-3-Lee-Yang-Parr[7] (B3LYP) hybrid density functional were employed for all calculations. London dispersion forces were modeled using the DFT-D3 dispersion method,[8] and basis set superposition error (BSSE) was dynamically accounted for through the geometrical counterpoise (gCP) scheme.[9] Vibrational normal mode eigenvectors and eigenvalues were calculated numerically within the harmonic approximation, and infrared intensities were determined using the Berry phase method.[10-12] The vibrational force constants and reduced masses were extracted from the mass-weighted Hessian matrix as described in detail elsewhere.[13] The energy convergence criteria were set to $\Delta E \leq 10^{-8}$ and 10^{-10} hartree for the optimization and frequency calculations, respectively.

B. PP-I Structure Determination Details

The structure of PP-I was determined by first building initial models using the previously published interatomic distances and angles,[14] followed by arranging the resulting helices in various configurations. The proposed geometry solutions were optimized using solid-state DFT without symmetry constraints, other than the translational symmetry of the periodic model. Upon complete relaxation, only two structures were energetically feasible, with the major difference between them being the parallel or antiparallel arrangements of neighboring polyproline helices as packed in the crystals. The polyproline strands exhibit a net dipole oriented along the helical axis, and while an antiparallel orientation of the adjacent dipoles may suggest a more stable crystal structure, the large diameter of the helices negates a large portion of that stabilization

energy. The preference for the all-parallel arrangement is related to it being able to pack together more efficiently, leading to a larger stabilization by London dispersion forces. Beyond energies, the calculated PXRD patterns of the two potential forms showed that the parallel structure was clearly in superior agreement with the experimental PXRD pattern (**Figure S.2**).

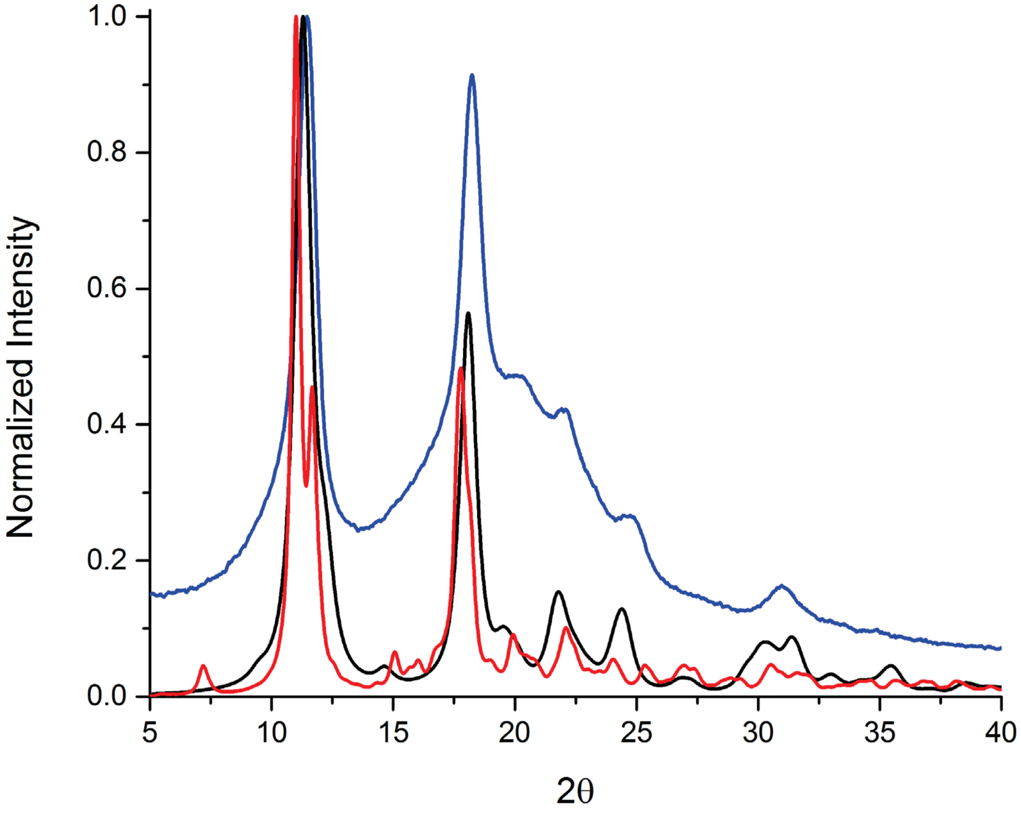

Figure S.2. Experimental PXRD pattern of PP-I (blue) with the accurate helical orientation (black) and incorrect orientation (red) patterns shown.

C. Gibbs Free Energy Curves

The solid-state vibrational calculations can be used to determine the temperature-dependent Gibbs free energy curves for the two forms, with the intersection of the two curves representing the transition temperature for phase stability. Plots of the Gibbs free energy for the two poly-L-proline helices are shown in **Figure S.3**, and the crossing temperature was found to be 557.39 K, nearly identical (yet slightly higher) to the experimentally determined decomposition temperature of polyproline (553 K). This result is consistent with the two forms not being able to undergo isomerization in the solid-state, and that such change is only accessible via solution phase processes. However those studies also found that these barriers could be lowered through external stabilization by solvent interactions, specifically related to the PP-II geometry offering greater hydrogen bond acceptor accessibility compared to the tightly wound PP-I.[15] Here, the energy analysis showed that PP-I is stabilized internally to a larger degree than PP-II by London dispersion forces (~16% larger London dispersion energies than PP-II) while PP-II is stabilized greater externally by both dispersion and dipole-dipole interactions (~6% and 17%, respectively).

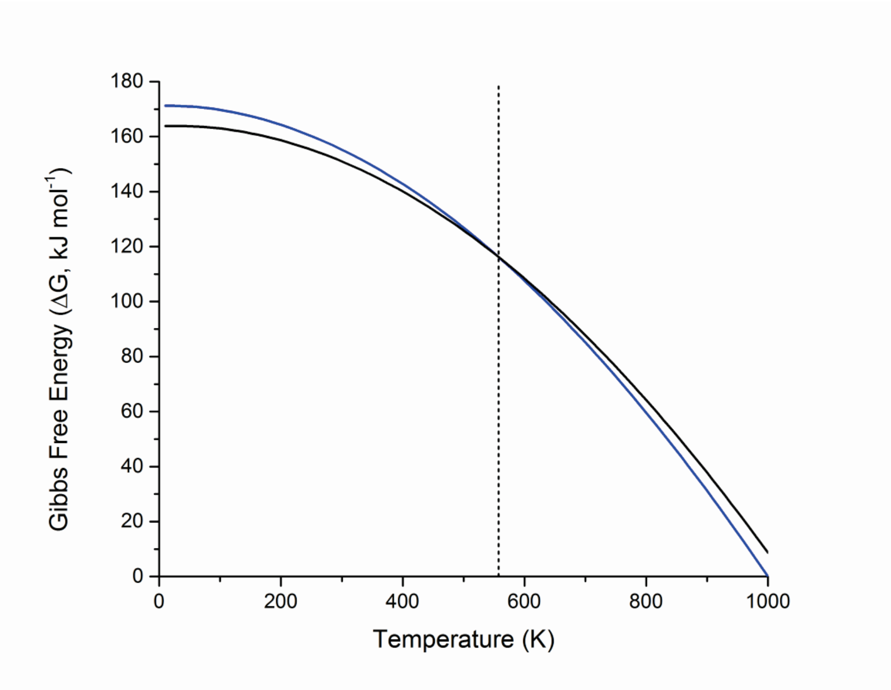

Figure S.3. Gibbs free energy curves for PP-I and PP-II (blue and black, respectively). The dashed line represents the temperature where the two curves intersect.

D. Spectral Deconvolution Analysis Results

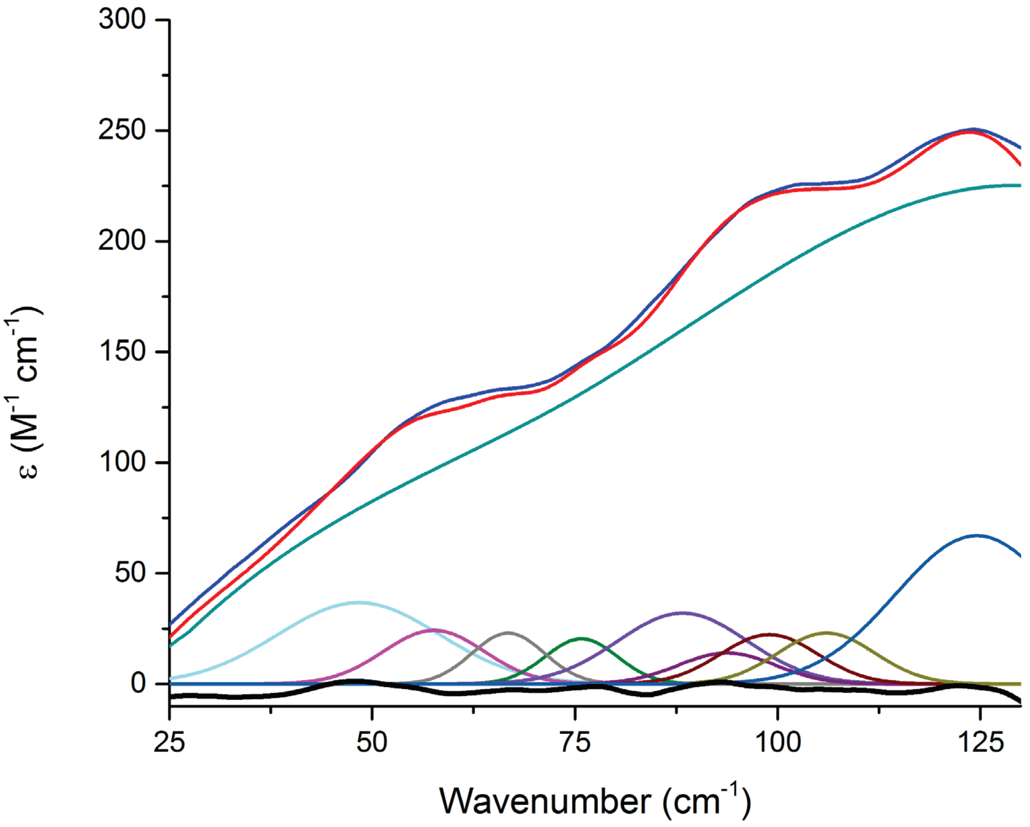

Figure S.4. Spectral deconvolution result of PP-I. The experimental spectrum (blue), result of calculated fit (red), baseline (green), observed-minus-calculated residual (black) and primitive functions (remainder) are shown.

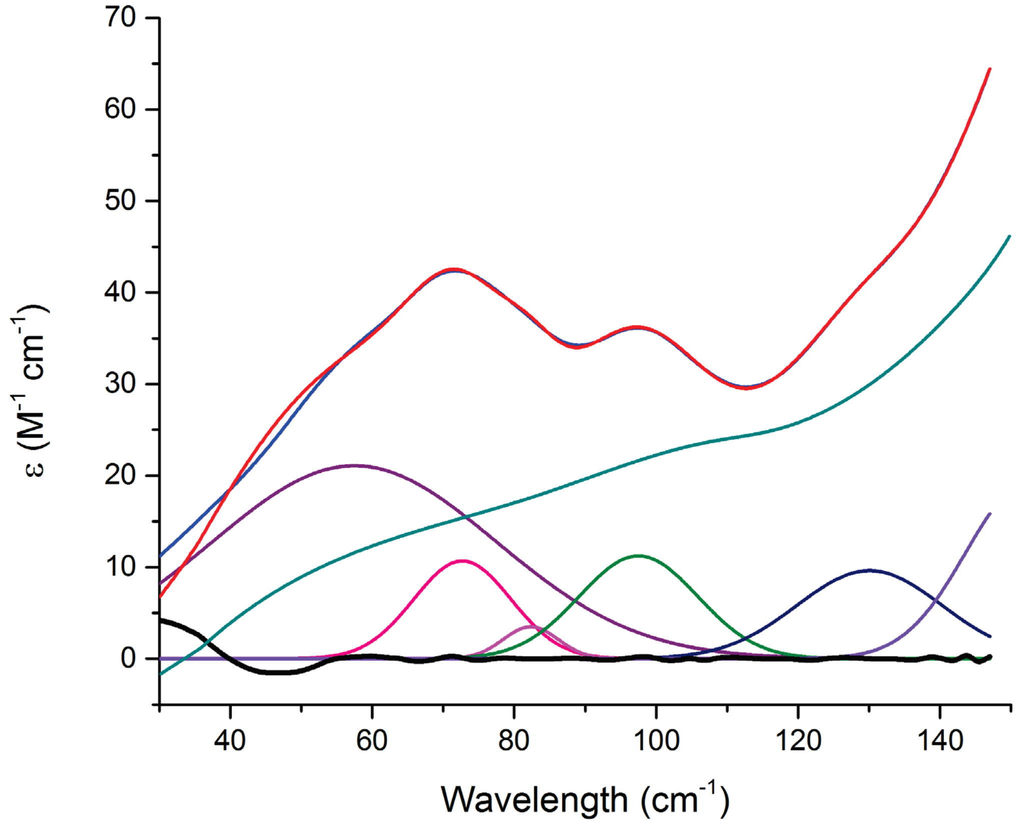

Figure S.5. Spectral deconvolution result of PP-II. The experimental spectrum (blue), result of calculated fit (red), baseline (green), observed-minus-calculated residual (black) and primitive functions (remainder) are shown.

Table S.1 Frequencies (cm^{-1}) of the observed absorptions in the spectra of PP-I and PP-II.

PP-I	PP-II
26.3	72.7
30.7	82.3
48.3	98.1
57.6	130.1
66.6	152.4
75.8	
77.2	
88.3	
93.7	
99.0	
106.0	

Table S.2. DFT-calculated IR-active frequencies (cm^{-1}) and intensities (km/mol) for PP-I

Frequency	Intensity	Frequency	Intensity	Frequency	Intensity	Frequency	Intensity
9.41	0.01	168.59	0.50	509.27	24.90	858.16	15.75
18.26	0.89	169.58	12.18	510.13	17.30	858.98	14.53
25.43	5.60	172.46	30.27	526.29	26.27	859.05	14.30
27.57	2.02	185.88	37.39	527.02	26.90	862.08	10.98
33.59	1.28	189.30	1.14	545.18	26.06	863.34	16.98
38.59	12.07	203.68	1.21	545.86	14.45	865.14	1.47
40.30	6.01	206.13	168.29	560.89	1.55	865.58	195.91
46.73	0.86	210.14	7.96	565.10	849.84	873.12	63.76
52.19	1.81	212.85	1.55	566.69	0.43	874.88	262.05
55.24	30.94	226.81	6.99	577.97	254.12	874.89	8.40
59.96	3.58	228.33	2.43	578.91	2.60	875.89	19.70
62.78	30.90	236.34	1.94	582.53	14.14	879.26	20.42
63.06	2.34	238.93	7.44	582.81	165.35	880.25	8.64
68.15	0.20	279.93	2.85	588.38	4.08	887.85	5.07
74.01	0.01	282.66	369.34	589.84	58.65	887.86	79.75
74.06	6.29	282.96	18.64	592.63	2.75	895.70	297.89
77.78	1.40	285.31	250.66	594.84	195.46	896.67	18.15
82.37	6.38	287.52	13.83	603.08	167.19	898.04	14.95
89.09	56.16	289.76	24.19	606.38	2.46	900.07	11.87
96.61	8.68	296.08	11.24	659.72	692.76	901.78	3.86
99.48	14.50	298.21	3.76	675.24	0.00	901.85	1.28
102.33	32.79	303.36	19.82	678.96	0.63	902.23	14.92
103.00	2.28	308.45	1428.71	696.82	1.57	906.12	1.41
103.72	11.83	312.72	615.09	698.81	8.93	907.12	51.18
107.12	6.53	318.82	8.76	713.52	32.53	909.43	2.17
111.23	18.62	319.96	128.31	717.78	37.64	924.95	1.78
112.60	2.29	322.91	60.75	724.47	50.96	925.25	21.98
114.10	0.80	330.33	54.77	728.94	545.97	927.10	2.85
114.80	8.60	345.80	19.28	730.72	2.83	927.47	49.20
116.84	12.54	350.34	17.22	745.35	174.89	930.65	1.89
120.51	2.89	362.71	0.22	745.79	2.16	931.91	139.84
123.18	46.57	364.76	613.83	749.52	3.03	932.55	2.44
124.60	34.75	366.22	2.61	751.63	19.54	933.29	5.74
128.82	2.31	375.66	4.10	753.35	17.48	936.54	93.21
131.02	8.84	375.89	330.46	762.12	52.07	936.84	8.59
131.31	6.38	380.32	2.67	765.70	70.01	966.32	9.38
133.43	0.01	382.30	114.16	777.36	27.25	966.78	1482.75
139.52	0.01	385.48	4.86	782.24	96.32	967.77	326.70
140.63	0.98	386.74	105.73	788.59	5.27	968.27	140.36
141.77	1.29	387.91	33.83	831.54	3025.60	969.77	13.32
158.37	122.28	389.64	10.70	850.04	8.39	972.41	21.53
160.94	11.86	392.69	104.25	852.75	0.06	973.63	30.02
161.39	22.43	393.26	29.91	854.08	2.99	975.63	14.85
164.05	365.31	503.23	0.51	857.15	107.13	977.46	0.39

Frequency	Intensity	Frequency	Intensity	Frequency	Intensity	Frequency	Intensity
977.99	2.03	1196.94	23.26	1324.71	227.26	1464.00	5.43
996.74	9.41	1205.45	26.47	1325.94	2.26	1465.42	14.89
998.16	239.49	1208.60	73.06	1326.29	482.33	1466.00	50.78
999.44	45.52	1209.29	66.59	1331.94	2336.42	1467.29	217.97
1000.11	1.52	1211.17	19.16	1339.12	25.67	1467.68	39.24
1005.54	40.31	1212.73	4.54	1341.17	32.76	1470.13	234.19
1006.24	132.54	1214.00	12.12	1342.99	0.25	1472.30	189.61
1009.70	390.77	1217.71	64.56	1344.25	77.71	1472.45	23.66
1013.49	7.50	1218.14	56.43	1345.24	24.91	1472.95	18.43
1015.05	1.27	1218.80	17.13	1345.69	25.11	1473.91	0.67
1021.71	1078.09	1219.80	4.21	1347.47	41.99	1474.70	443.11
1052.32	22.67	1220.34	50.69	1349.29	94.02	1476.32	66.98
1052.94	130.82	1220.60	362.03	1350.74	68.02	1476.59	3.22
1055.88	5.40	1222.85	101.32	1356.02	388.74	1478.04	3.10
1056.49	2.76	1223.18	290.01	1356.48	76.20	1481.99	12.12
1065.60	40.68	1226.46	168.98	1357.58	45.21	1486.04	30.28
1066.03	3.17	1226.92	58.26	1358.18	12.18	1488.01	2.72
1066.96	44.96	1247.43	54.75	1359.07	8.66	1490.29	5.84
1069.62	189.41	1247.90	213.40	1359.61	18.90	1491.14	17.78
1072.92	41.47	1248.48	45.92	1360.19	11.91	1494.65	11.78
1073.72	0.87	1250.09	33.46	1361.17	46.07	1496.62	58.94
1103.20	1.35	1252.36	1.23	1365.18	283.13	1499.66	22.77
1103.32	78.22	1253.09	12.39	1367.26	44.39	1500.45	7.62
1104.16	0.65	1258.06	21.07	1373.00	217.04	1501.60	22.94
1104.62	1.01	1260.44	230.81	1375.43	19.21	1501.99	19.04
1105.25	1.24	1260.58	19.27	1379.23	1.88	1502.93	23.45
1105.98	0.31	1262.17	17.71	1380.34	206.85	1504.37	4.65
1107.81	127.99	1282.71	108.11	1381.62	137.99	1504.73	1.97
1108.48	3.07	1284.35	2.94	1384.92	59.57	1540.91	14431.75
1109.86	21.53	1287.21	1.93	1386.66	55.11	1706.02	25.49
1110.24	2.59	1288.52	42.06	1390.88	5.31	1706.45	416.36
1171.65	5.61	1288.99	1.84	1398.12	7.53	1709.64	468.84
1173.06	45.07	1292.36	22.98	1409.52	3376.50	1711.28	71.06
1176.35	11.35	1293.85	23.83	1436.24	59.58	1714.17	76.17
1177.20	270.82	1295.45	368.55	1443.15	52.40	1714.38	5122.83
1178.58	6.47	1298.78	261.98	1449.20	54.68	1724.79	91.86
1179.09	36.46	1299.43	3.24	1450.73	57.39	1726.36	234.95
1180.37	52.55	1308.95	9425.95	1452.03	19.65	1739.88	15.24
1182.23	41.71	1315.12	3.86	1454.64	320.00	1743.28	0.81
1187.21	21.31	1319.16	1600.72	1454.78	208.32	2984.63	205.36
1187.50	0.43	1320.32	4.71	1456.18	1.98	2985.93	697.49
1192.02	8.99	1321.77	4.65	1458.71	3.04	3008.53	131.38
1192.21	0.82	1321.90	391.89	1458.87	343.00	3009.20	383.24
1195.56	10.51	1324.20	0.52	1461.99	35.96	3012.47	198.51

Frequency	Intensity	Frequency	Intensity
3014.58	172.13	3110.63	15.09
3026.17	7.81	3114.80	40.40
3027.97	92.02	3115.32	31.74
3036.81	33.89	3117.36	729.59
3037.89	743.15	3117.86	17.75
3040.26	161.19	3119.21	1157.47
3041.63	60.53	3119.36	18.83
3041.74	16.64	3121.94	842.72
3044.88	26.99	3123.61	1.06
3046.02	7.19	3129.09	2392.65
3046.72	38.04	3131.19	35.95
3052.56	35.64	3133.01	2296.17
3052.67	124.66	3135.80	56.06
3056.35	120.41	3136.25	35.61
3057.34	73.43	3139.74	4765.00
3058.75	15.27	3141.34	1.56
3059.92	75.96	3143.73	41.62
3063.49	1.34	3144.54	2889.14
3063.55	82.61	3154.27	5.42
3065.08	11.22	3162.77	18.35
3065.68	235.49	3209.25	1.00
3070.32	19.97		
3070.32	47.48		
3074.91	20.72		
3075.99	193.77		
3077.20	46.33		
3078.36	99.27		
3081.61	75.84		
3083.19	37.76		
3086.64	34.85		
3087.23	282.01		
3088.19	133.01		
3090.30	60.14		
3091.24	2.94		
3092.03	5.74		
3094.67	0.03		
3095.25	12.48		
3099.90	35.87		
3101.04	1197.35		
3102.14	39.45		
3105.96	24.66		
3106.29	829.14		
3108.55	54.27		
3110.33	13.51		

Table S.3. DFT-calculated IR-active frequencies (cm^{-1}) and intensities (km/mol) for PP-II

Frequency	Intensity	Frequency	Intensity
55.75	11.35	1196.38	16.90
74.67	1.55	1200.46	31.00
75.24	16.83	1226.02	186.19
100.10	15.67	1227.37	28.21
126.35	2.68	1256.04	1.16
131.68	7.82	1257.75	0.12
144.49	240.40	1279.67	82.16
170.75	0.85	1288.59	99.33
190.25	0.22	1325.56	20.11
191.24	11.49	1329.48	4.93
226.10	2.84	1340.74	82.80
260.22	7.59	1346.85	91.99
321.43	26.98	1351.89	14.15
350.02	2.65	1363.05	48.81
380.53	13.34	1368.20	4.96
426.93	44.32	1378.46	64.23
508.12	0.00	1449.13	640.65
550.91	32.34	1454.40	249.14
590.12	1.81	1469.05	185.03
607.09	2.32	1470.76	84.77
691.61	85.33	1475.32	36.42
722.45	34.83	1480.07	88.44
761.19	0.04	1500.80	0.87
777.32	26.27	1508.19	0.35
844.01	46.54	1710.66	2044.30
850.58	38.40	1724.21	186.34
865.50	21.19	3026.15	41.59
869.26	5.57	3029.16	114.92
912.06	11.04	3060.60	16.42
915.65	36.71	3061.95	23.82
936.62	1.04	3075.29	119.06
937.46	6.28	3075.40	23.11
966.19	17.29	3078.45	14.84
995.04	2.33	3081.07	97.24
1011.76	19.18	3094.88	34.30
1033.96	16.09	3095.25	1.03
1056.62	10.61	3114.20	115.33
1070.03	1.77	3123.22	6.03
1104.97	7.23	3141.21	10.34
1112.70	0.00	3142.40	7.93
1178.85	1.24		
1183.30	89.41		

E. PP-I CIF

```
data_PPF1
_symmetry_cell_setting          monoclinic
_symmetry_space_group_name_H-M   'P 21'
_symmetry_Int_Tables_number      4
loop_
_symmetry_equiv_pos_site_id
_symmetry_equiv_pos_as_xyz
1 x,y,z
2 -x,1/2+y,-z
_cell_length_a                9.14900228
_cell_length_b                18.92718359
_cell_length_c                9.20299131
_cell_angle_alpha              90.000000
_cell_angle_beta              59.278284
_cell_angle_gamma              90.000000
_cell_volume                  1369.98
loop_
_atom_site_label
_atom_site_type_symbol
_atom_site_fract_x
_atom_site_fract_y
_atom_site_fract_z
C C 0.8276375452806 0.43245863269917 1.07427969807817
C C 1.1671727700399 0.2364208856348 0.998002896864204
C C 0.94135138124756 0.0363137301487 0.91112132967896
C C 1.05480162801772 0.3304200868807 0.8175092825812
C C 0.8730866036357 0.1408632077258 1.1801370574150
C C 0.90449522689719 0.44795530467499 0.8832043727942
C C 1.01309923830554 0.2569669674838 1.1726234188941
C C 1.1357585082993 0.0501762507332 0.8196365371131
C C 1.2138516564414 0.3482577722380 0.8297272650316
C C 0.8240883632562 0.1553606442864 1.04477703981188
O O 0.8779139810799 0.504364611227709 0.8337901888258
O O 1.007357712829828 0.3147227104041 1.2375942732200
O O 1.1972317075893 0.1055625213712 0.7413537947221
O O 1.2940622773011 0.40336516104575 0.7691227599336
O O 0.7576595269640 0.2119922932710 1.04396415182152
N N 0.992661556528396 0.3943396426017 0.7761453666150
N N 0.8894273811567 0.2076293616559 1.2527616798926
N N 0.7665455606165 0.4976236786232 1.1746557427575
N N 1.2630678468038 0.2993546429568 0.90427209694193
N N 0.8481418128764 0.1026705815847 0.93494018386091
C C 1.09923473109778 0.2821139015106 0.6644808310625
C C 0.7235817463467 0.1037425832480 1.3355964993496
C C 0.6621439692241 0.3885630271675 1.1420186974714
C C 1.3011050899917 0.1946003686335 1.01896032453379
C C 0.8942787845655 -0.007419581428498 0.7981922966570
C C 1.1686404704888 0.3342757636982 0.5157098721598
```

C C 0.6097269827730 0.1659329580773 1.4397686440886
C C 0.5277699333729 0.4457483447857 1.1813635139110
C C 1.4075526005321 0.2530739961393 1.03693073969087
C C 0.8763183854358 0.0492526590203 1 0.6884910550431
C C 1.06257757039571 0.40177366346472 0.5929116876803
C C 0.7385961984475 0.2227019914435 1.4199119972537
C C 0.5802469223522 0.5053629586346 1.2585153424986
C C 1.4165741988589 0.3109233383060 0.91544155247017
C C 0.7939694832097 0.1116434956715 0.8100962175013
H H 1.1391228468087 0.45043504245328 0.5464530353972
H H 0.6941232520670 0.2773048645563 1.4243352690186
H H 0.5470241117752 0.5584338309247 1.2348769363485
H H 1.4173782973361 0.3650145672824 0.95986522580201
H H 0.8344564791221 0.1635532200313 0.7474565633186
H H 0.95745415557583 0.40645148287619 0.5669565803631
H H 0.7727737904387 0.2184212813001 1.5192538727143
H H 0.5214303590035 0.4990176415700 1.3970202966142
H H 1.5315238908982 0.3059264245446 0.7879992064182
H H 0.6533022220162 0.1098829780856 0.8751978179397
H H 1.3044669746993 0.3447671976090 0.4678708896482
H H 0.5343268071703 0.1847919891317 1.3845201944830
H H 0.5359324682274 0.4641320931717 1.06343666614977
H H 1.3416415298372 0.2733155449394 1.1677181506404
H H 1.002192181090264 0.06409876227970 0.5809843162308
H H 1.1559171005692 0.3148248423114 0.4098883113729
H H 0.3971186266447 0.4272209494475 1.2671257906332
H H 1.5339609950563 0.2349775148737 1.008158011111633
H H 0.7983098727869 0.03146661563685 0.6357404514697
H H 1.1871370732076 0.2387963963096 0.6475208156602
H H 0.6587682835822 0.06413846292927 1.3002134291012
H H 0.6709913335894 0.3480902732780 1.05202403406696
H H 1.2439650593403 0.1568681815720 1.1252043819232
H H 0.98774111826230 -0.04895049062403 0.7253398154170
H H 0.97995820897862 0.2577171013600 0.6863822499552
H H 0.7754642143141 0.07563573546941 1.4047044350839
H H 0.6359114172256 0.3606640031814 1.2584519224108
H H 1.3790654215499 0.1635289845748 0.90257541030963
H H 0.7717823959523 -0.03417715612880 0.8806352177964
H H 0.91921161524663 0.40492833227900 1.09849228880581
H H 1.1275285402105 0.2053877508637 0.92285476034934
H H 0.8954357390236 0.0094343162874 1.03313286726151
H H 0.95411620870060 0.3058475135279 0.93449617466033
H H 0.98969352388348 0.1088941502267 1.1300536110441
H H 0.5186019327900 0.1521014462489 1.5723431447493

#END

F. PP-II CIF

```
data_PPFII
_symmetry_cell_setting           trigonal
_symmetry_space_group_name_H-M   'P 32'
_symmetry_Int_Tables_number      145
loop_
_symmetry_equiv_pos_site_id
_symmetry_equiv_pos_as_xyz
1 x,y,z
2 -y,x-y,2/3+z
3 -x+y,-x,1/3+z
_cell_length_a                6.64326727
_cell_length_b                6.64326727
_cell_length_c                9.60125787
_cell_angle_alpha              90.000000
_cell_angle_beta               90.000000
_cell_angle_gamma              120.000000
_cell_volume                  366.963
loop_
_atom_site_label
_atom_site_type_symbol
_atom_site_fract_x
_atom_site_fract_y
_atom_site_fract_z
C C 0.01596654385846 -0.007103170474542 0.09395528235756
N N -0.02686067948053 -0.1706193665484 0.1922607731086
O O -0.07315512385516 0.1170986949121 0.09740575607850
C C 0.1951099771137 0.02527503386097 -0.01941264119432
C C 0.4441082003882 0.1959364471137 0.03256508878296
C C 0.4757473164179 0.4361945058927 -0.003679655052814
C C 0.06113485586220 -0.3355421149927 0.1944405614581
H H 0.2532290052619 -0.2436429620236 0.1939473463811
H H -0.0007723785251607 -0.4512033161828 0.1025257418070
H H 0.5704872176947 0.1631686350255 -0.02261389932837
H H 0.4650527450147 0.1748761110167 0.1445206847866
H H 0.1728693246817 -0.1441752125955 -0.05097171699718
H H 0.65859204844703 0.5699140762710 -0.0194662682060
H H 0.40430435200609 0.4956067996423 0.0803144126849
N N 0.1706193665484 0.14375868706787 -0.1410725602247
C C -0.16983494325273 -0.1951099771137 0.31392069213901
C C -0.0395528105252 -0.4757473164179 0.329653678280519
C C 0.3355421149927 0.39667697085490 -0.1388927718752

#END
```

G. References

(1) Version 2011.8-0 ed.; Bruker-AXS Inc.: Madison, WI, 2011, 2011.

(2) Macrae, C. F.; Bruno, I. J.; Chisholm, J. A.; Edgington, P. R.; McCabe, P.; Pidcock, E.; Rodriguez-Monge, L.; Taylor, R.; van de Streek, J.; Wood, P. A. *J. Appl. Crystallogr.* **2008**, *41*, 466.

(3) Huang, N. E.; Shen, Z.; Long, S. R.; Wu, M. C.; Shih, H. H.; Zheng, Q.; Yen, N.-C.; Tung, C. C.; Liu, H. H. *Proc. R. Soc. London, Ser. A* **1998**, *454*, 903.

(4) Battista, B. M.; Knapp, C.; McGee, T.; Goebel, V. *GEOPHYSICS* **2007**, *72*, H29.

(5) Dovesi, R.; Orlando, R.; Erba, A.; Zicovich-Wilson, C. M.; Civalleri, B.; Casassa, S.; Maschio, L.; Ferrabone, M.; De La Pierre, M.; D'Arco, P.*et al. Int. J. Quantum Chem* **2014**, *114*, 1287.

(6) Weigend, F.; Ahlrichs, R. *Phys. Chem. Chem. Phys.* **2005**, *7*, 3297.

(7) Becke, A. D. *J. Chem. Phys.* **1993**, *98*, 5648.

(8) Grimme, S.; Antony, J.; Ehrlich, S.; Krieg, H. *J. Chem. Phys.* **2010**, *132*, 154104.

(9) Brandenburg, J. G.; Alessio, M.; Civalleri, B.; Peintinger, M. F.; Bredow, T.; Grimme, S. *J. Phys. Chem. A* **2013**, *117*, 9282.

(10) Noel, Y.; Zicovich-Wilson, C. M.; Civalleri, B.; D'Arco, P.; Dovesi, R. *Phys. Rev. B.* **2001**, *65*, 014111.

(11) Pascale, F.; Zicovich-Wilson, C. M.; López Gejo, F.; Civalleri, B.; Orlando, R.; Dovesi, R. *J. Comput. Chem.* **2004**, *25*, 888.

(12) Zicovich-Wilson, C. M.; Pascale, F.; Roetti, C.; Saunders, V. R.; Orlando, R.; Dovesi, R. *J. Comput. Chem.* **2004**, *25*, 1873.

(13) Ochterski, J. W., Vibrational analysis in Gaussian **1999**.

(14) Traub, W.; Shmueli, U. *Nature* **1963**, *198*, 1165.

(15) Moradi, M.; Babin, V.; Roland, C.; Darden, T. A.; Sagui, C. *PNAS* **2009**, *106*, 20746.
